# From Upconversion Nanoparticles to Proteins: Probing Hydration-Water Density Fluctuations by Luminescence Thermometry

**DOI:** 10.1021/acs.accounts.5c00883

**Published:** 2026-03-13

**Authors:** Ramon S. Raposo Filho, Yongwei Guo, Fernando E. Maturi, Carlos D. S. Brites, Luís D. Carlos

**Affiliations:** Phantom-g, CICECO—Aveiro Institute of Materials, Physics Department, 56062 University of Aveiro, 3810-193, Aveiro, Portugal

## Abstract

Water appears simple, yet its anomalous behavior reveals an unexpected structural complexity. A growing body of evidence indicates that many of water’s anomalies arise from fluctuations between low-density (LD) and high-density (HD) local structural motifs, a form of polymorphism that is well established in the supercooled regime and increasingly supported at ambient conditions. Yet, how these structural motifs manifest within hydration layers, where water interacts with nanoparticles, proteins, and charged interfaces, remains far less understood. This interfacial water governs colloidal stability, biomolecular function, and chemical reactivity, but its microscopic organization is difficult to probe directly with conventional bulk techniques.

In this Account, we describe how luminescence nanothermometry provides a powerful and versatile approach to accessing density fluctuations in the hydration layer. By monitoring temperature-dependent optical and Brownian observables of luminescent probes, structural reorganizations of the surrounding hydration layer can be inferred with nanoscale sensitivity. Over the past several years, our group has shown that lanthanide-doped upconversion nanoparticles (UCNPs) and fluorescent proteins, such as enhanced green fluorescent protein (EGFP), act as local reporters of hydration-water density fluctuations.

A central observation emerging from these studies is the existence of a crossover temperature, *T*_c_, at which hydration-water observables exhibit bilinear temperature dependencies. This *T*_c_ correlates with the depletion of LD motifs in the hydration shell and typically falls within the 315–330 K range, close to the minimum of water’s isothermal compressibility. Importantly, *T*_c_ depends on the nature of the probe and its interaction with the surrounding water.

By systematically varying nanoparticle size, pH, surface chemistry, and probe type, we show that previously contradictory trends in *T*_c_ can be unified by a single parameter: the effective surface charge density of the probe. When *T*_c_ is plotted against this quantity, data from UCNPs with different sizes and surface functionalizations, as well as from fluorescent proteins at different concentrations, collapse onto a master curve. This result demonstrates that interfacial electrostatics govern the stability of LD motifs in the hydration layer, providing a physically intuitive framework that links nanoscale charge distributions to local water structure.

We further extend this framework by examining nuclear quantum effects through isotopic substitution. Using EGFP as a model biomolecular probe, we show that replacing H_2_O with D_2_O shifts *T*_c_ upward by ≈10 K and enhances protein thermal stability, consistent with stronger hydrogen bonding and the displacement of thermodynamic anomalies in heavy water. In contrast, several inorganic and molecular probes fail to resolve a comparable isotopic shift, highlighting that the detectability of LD/HD fluctuations might be probe-dependent. Control experiments in H_2_^18^O confirm that hydrogen, rather than oxygen, dominates these quantum effects.

Together, these results establish luminescent nanoprobes as sensitive reporters of hydration-water density fluctuations and reveal how interfacial charge, confinement, and quantum effects sculpt water structure at the nanoscale. Beyond resolving long-standing questions about water’s anomalies, this approach opens new avenues for understanding protein stability, designing functional nanomaterials, and exploiting hydration-water density fluctuations in chemical and biological systems.

## Graphical abstract

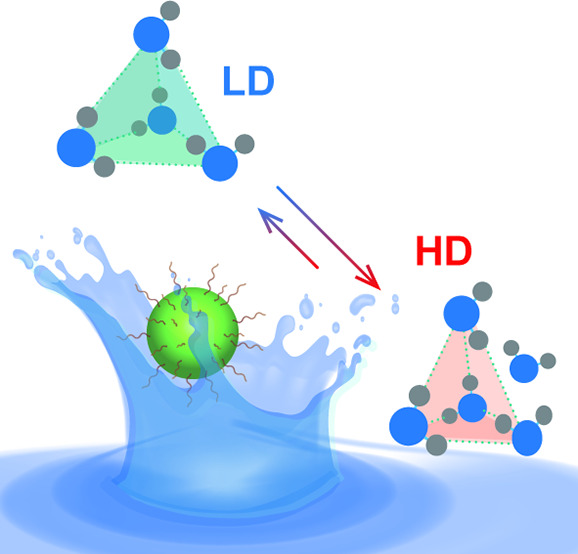

## Key References

-

Brites, C. D. S. ; Zhuang, B. L. ; Debasu, M. L. ; Ding, D. ; Qin, X. ; Maturi, F. E. ; Lim, W. W. Y. ; Soh, D. ; Rocha, J. ; Yi, Z. G. Decoding a percolation phase transition of water at similar to 330 K with a nanoparticle ruler. J. Phys. Chem. Lett. 2020, 11 (16), 6704–6711 32672973 10.1021/acs.jpclett.0c02147.^1^ First experimental evidence that the Brownian dynamics of NaYF_4_:Yb/Er upconversion nanoparticles (UCNPs) sense a structural transition in water near 330 K. Temperature-dependent Brownian-velocity measurements revealed a percolation-like onset of fluctuations between low-density (LD) and high-density (HD) water motifs, establishing the structural crossover temperature (*T*_c_) as a meaningful physical descriptor for hydration water.
-

Maturi, F. E. ; Raposo Filho, R. S. ; Brites, C. D. S. ; Fan, J. ; He, R. ; Zhuang, B. ; Liu, X. ; Carlos, L. D. Deciphering density fluctuations in the hydration water of Brownian nanoparticles via upconversion thermometry. J. Phys. Chem. Lett. 2024, 15 (9), 2606–2615 38420927 10.1021/acs.jpclett.4c00044PMC10926164.^2^ Demonstrated that the crossover temperature *T*_c_ depends on nanoparticle size and pH. Upconversion nanothermometry revealed that nanoscale confinement and interfacial chemistry modulate the fluctuations between LD and HD motifs in the hydration layer, indicating that *T*_c_ is a probe-dependent, rather than a universal, property.
-

Guo, Y. W. ; Maturi, F. E. ; Brites, C. D. S. ; Carlos, L. D. Exploring green fluorescent protein Brownian motion: Temperature and concentration dependencies through luminescence thermometry. Adv. Phys. Res. 2024, 3 (11), 2400085 .^3^ Extended luminescence nanothermometry to biomolecules by quantifying the Brownian motion of enhanced green fluorescence protein (EGFP). Temperature- and concentration-dependent fluctuations in protein hydration water were shown to regulate EGFP dynamics, illustrating how LD/HD structural motifs modulate biomolecular environments.
-

Raposo Filho, R. S. ; Brites, C. D. S. ; He, R. ; Liu, X. ; Carlos, L. D. Thermal diffusivity of nanofluids: A simplified temperature oscillation approach. Phys. Fluids 2025, 37 (2), 022027 .^4^ Provided control experiments confirming that *T*_c_ is not an artifact of bulk nanofluid thermal properties. Thermal diffusivities of UCNP nanofluids were shown to be indistinguishable from that of pure water, confirming the interpretation that *T*_c_ reflects hydration-layer structural fluctuations.
-

Raposo Filho, R. S. ; Brites, C. D. S. ; Carlos, L. D. Tuning water density fluctuations with surface-charged colloidal nanoparticles probed by luminescence. J. Phys. Chem. Lett. 2025, 16, 11683–11689 41172297 10.1021/acs.jpclett.5c02981.^5^ Identified the effective surface charge density as a unifying parameter governing *T*_c_. By collapsing size- and pH-dependent data onto a single master curve, this work established interfacial electrostatics as the primary factor controlling LD-motif stability.
-

Guo, Y. ; Maturi, F. E. ; Raposo Filho, R. S. ; Brites, C. D. S. ; Carlos, L. D. Exploring water beyond the solvent: Insights into density fluctuations and EGFP unfolding via luminescence thermometry. J. Phys. Chem. B 2025, 129 (46), 12042–12050 41196670 10.1021/acs.jpcb.5c06143PMC12772516.^6^ Linked fluctuations between LD and HD motifs in hydration water to protein unfolding. Using EGFP in H_2_O and D_2_O, this study demonstrated that *T*_c_ shifts with isotopic composition and correlates with protein stability, establishing a direct link between hydration water structural fluctuations and biomolecular function.

## 1 Introduction

Liquid water, the principal constituent of the human body and the most abundant liquid on Earth, plays a vital role across natural and technological processes.^7–12^ Despite its apparent simplicity, water displays more than 60 anomalous properties relative to most liquids.^13–17^ A first class of anomalies, including its high boiling point, melting point, and surface tension, arises from the strength and directionality of hydrogen bonding, as also observed in other strongly hydrogen-bonded liquids (e.g., HF and NH_3_ ^18,19^ ). A second class of anomalies, however, points toward deeper structural heterogeneity in the liquid. These include a minimum in isobaric heat capacity at 308 K, a negative thermal expansion coefficient below 277 K, and a minimum in isothermal compressibility at 319 K.^15^ The anomalies have been investigated across different length scales using distinct experimental techniques (Figure 1).

**Figure 1:**
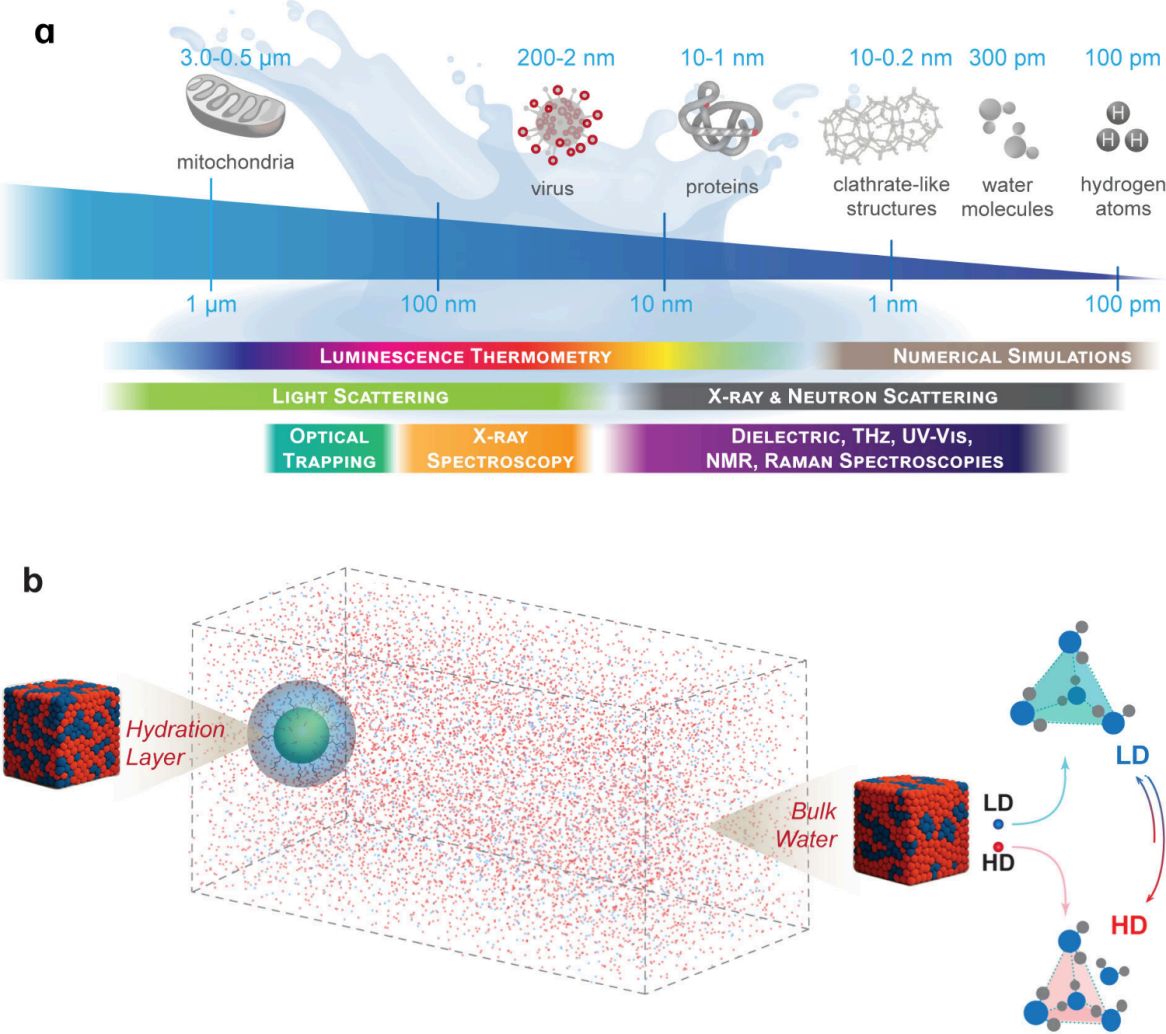
(a) Infographic summarizing the experimental techniques used to investigate anomalies in liquid water across multiple length scales. (b) Schematic illustrating how a luminescent nanoprobe (here represented by a UCNP) senses LD (blue dots) and HD (red dots) structural motifs in its hydration layer (shadowed blue region), where LD motifs are less frequent than in bulk water (see the discussion in section 4). Right panel: Temperature-dependent LD/HD fluctuations, with LD motifs disappearing for *T* > *T*_c_. Adapted from ref 5. Copyright 2025 American Chemical Society.

Analogous anomalies in other tetrahedral liquids^20^ (e.g., Si,^21^ P,^22^ and S^23^) are well described by a phenomenological two-state framework, where directional bonding favors open, low-density (LD) networks that compete with more compact, high-density (HD) arrangements.^24,25^ The model further predicts a liquid–liquid critical point (LLCP) when cooperativity (representing the energetic gain associated with forming neighboring like-structured units) is sufficiently strong.^26,27^ First proposed by Poole et al.,^28^ the LLCP is now supported by extensive theoretical^27,29–33^ and experimental^34–45^ evidence. Below it, water separates into two macroscopic liquids: a low-density liquid (LDL) characterized by an open tetrahedral hydrogen-bond network, and a high-density liquid (HDL) with a more compact and disordered network stabilized by shorter, stronger hydrogen bonds.^14,15,19,46–52^ Above the LLCP, water exists as a single supercritical liquid composed of fluctuating LD and HD structural motifs^53^ (Figure 1b), with the amplitude and length scale of fluctuations decreasing as temperature or pressure increases.^14,54^

Evidence from scattering, spectroscopy, and simulations supports coexisting LD and HD motifs^40,53^ even at ambient conditions, but both the crossover temperature *T*_c_ marking the disappearance of LD motifs and the absolute LD fraction remain under debate (see Section 2).

Reported LD fractions span from ≈1% to 30% at room temperature, depending on the experimental method or model (see Table 1 of ref.^5^). Some studies report a gradual decrease in LD population with increasing temperature,^55–57^ whereas others identify a single transition temperature T* ^54,58^ (or *T*_c_) marking the loss of LD motifs. High-precision X-ray diffraction and small-angle X-ray scattering results locate T* (or *T*_c_) between 320 and 340 K,^19^ coinciding with the minimum in water’s isothermal compressibility.^59^ This temperature range thus appears as a thermodynamic fingerprint of structural crossover between LD and HD fluctuations.

**Table 1:** Crossover Temperature (*T*_c_) of the Water-Suspended Nanomaterials Shown in Figure 3, Determined As Detailed in Ref 2

Nanoprobe	Morphology	Size (nm)	*T*_c_ (K)
CdTe	Spherical	1.2	323 ± 6
GdVO_4_:Yb/Nd	Spherical	3.0	323 ± 3
Ag_2_S	Elliptical	5.9 × 7.5	322 ± 1
NaYF_4_:Yb/Er	Hexagonal	257.0 × 143.0	317 ± 3
NaYF_4_:Yb/Er	Spherical	15.0	331 ± 1
NaYF_4_:Lu/Yb/Er@SiO_2_	Spherical	100.0	329 ± 2

Although most research focuses on bulk water, far less is known about how LD and HD structural motifs manifest within hydration environments, where interactions with proteins, nanoparticles, ions, or charged interfaces strongly perturb local water structure. The hydration layer plays central roles in biochemical function, colloidal stability, and interfacial thermodynamics,^60–62^ yet its temperature-dependent structural fluctuations remain poorly understood. Because hydration water differs significantly from bulk water,^61,63–67^ probes capable of sensing their immediate local environment provide a unique opportunity to access the structural dynamics of the hydration water. Light-emitting nanoprobes, such as lanthanide-doped upconversion nanoparticles (UCNPs) and fluorescent proteins, are particularly attractive because their optical and Brownian signatures respond directly to local hydrogen-bond rearrangements in the hydration layer.

Within our group, luminescence nanothermometry has emerged as a powerful approach for probing the fluctuations between LD and HD motifs through temperature-dependent Brownian-velocity measurements of NaYF_4_:Yb/Er UCNPs^1,2,5^ and fluorescent proteins.^3,6^ Brites et al.^1^ first showed a percolation-like transition associated with the emergence of LD motifs in the hydration layer of the UCNPs. Maturi et al.^2^ later reported that *T*_c_ systematically depends on nanoparticle size and pH, whereas Raposo Filho et al.^5^ identified surface charge density as the primary factor governing this transition. Extending these ideas to biological systems, Guo et al.^6^ correlated *T*_c_ with EGFP unfolding, establishing a direct connection between hydration water local structure and protein stability. Complementary control experiments confirmed that these effects originate from structural fluctuations in hydration water, rather than changes in the bulk nanofluids’ thermal properties.^4^

By presenting these developments in chronological order, this Account traces the evolution of our contributions and demonstrates that luminescence nanothermometry provides a powerful means of understanding how water reorganizes within the hydration layer of light-emitting nanoprobes.

## 2 Low-Density and High-Density Structural Motifs in Bulk Water

One of the most direct experimental routes to interrogate the microscopic structure of liquid water is the oxygen–oxygen pair distribution function, *g*_OO_(*r*), which resolves the spatial correlations between neighboring molecules. High-energy X-ray diffraction measurements by Skinner et al.^59^ provided accurate *g*_OO_(*r*) curves over 254–366 K, enabling a detailed assessment of how the local structure of bulk water evolves with temperature. Their data reveal systematic shifts in the first two coordination shells above ≈320 K (Figure 2a, b), coinciding with the minimum in the isothermal compressibility (Figure 2c). These shifts reflect a progressive reduction of tetrahedral ordering and a corresponding increase in interstitial occupancy, capturing the continuous structural evolution as temperature increases.

**Figure 2:**
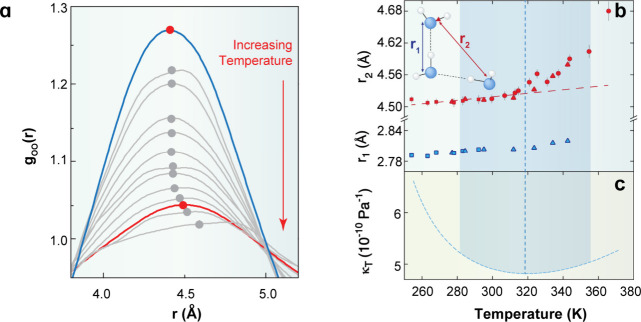
(a) Temperature-dependent evolution of the second peak of the oxygen–oxygen pair distribution function, *g*_OO_(*r*), reflecting the diminishing population of LD structural motifs in bulk water. The darker curves correspond to 254.2 and 342.7 K. (b) Positions of the first (*r*_1_) and second (*r*_2_) peak maxima in *g*_OO_(*r*), obtained by locally fitting Gaussian functions around each maximum. The uncertainty in *r*_1_ corresponds to the symbol size. (c) Isothermal compressibility of water between 260 and 375 K. The shaded region marks a 0.5% variation in the compressibility. Adapted with permission from ref 59. Copyright 2014 American Institute of Physics.

An increasing number of studies support the existence of two distinct local structural motifs, despite ongoing debate.^68–71^ In the supercooled regime, infrared spectroscopy provides direct evidence for the coexistence of low- and high-density configurations.^40^ At ambient conditions, signatures consistent with a two-state behavior have been revealed by a broad range of techniques, including X-ray scattering,^47^ X-ray absorption (XAS) and emission spectroscopies,^72^ high-precision density measurements,^54^ infrared^73^ and Raman^74^ spectroscopies, time-resolved optical Kerr effect measurements,^75^ and combined XAS and X-ray Raman experiments.^76^ Across these techniques, temperature-dependent spectral and scattering changes reflect variations in local hydrogen-bond topology. Molecular simulations further identify structural indicators of two-state behavior at ambient conditions,^53^ reinforcing the view that water’s anomalies arise from fluctuations between coexisting LD and HD motifs.

Bulk techniques, however, are intrinsically limited in resolving the nanometer-scale, subnanosecond fluctuations predicted by simulations. Below *T*_c_, LD motifs form transient domains embedded within a matrix of HD-like coordination.^54^ These motifs shrink with increasing temperature and disappear above *T*_c_, where HD motifs dominate the local structure. Because such fluctuations are both spatially and temporally heterogeneous, methods that average over large volumes or probe only global structure struggle to isolate their signatures.

This intrinsic multiscale heterogeneity motivates the use of nanoscale luminescent probes. Their optical and Brownian responses depend directly on the structure and dynamics of their hydration layer, making them uniquely sensitive to the evolving balance between LD and HD motifs. As we show in subsequent sections, luminescence nanothermometry exploits this sensitivity to reveal structural crossovers in hydration water surrounding nanoparticles and proteins (hydration layer), providing insights inaccessible to traditional ensemble-averaged techniques.

## 3 Hydration Water and the Crossover Temperature

Water in the vicinity of solutes, nanoparticles, or biomolecular surfaces is structurally and dynamically distinct from bulk water.^61,63–67,77,78^ The altered hydrogen-bond topology, modified local density, and restricted mobility within hydration shells give rise to structural fluctuations whose temperature dependence cannot be directly inferred from bulk measurements. Yet these interfacial environments govern biochemical activity, colloidal stability, and a wide range of aqueous processes, making it essential to determine how LD and HD structural motifs reorganize around dispersed probes.

A wide variety of water-dispersed systems, including quantum dots (QDs),^79^ plasmonic^80^ and luminescent nanoparticles,^81,82^ organic molecules,^83,84^ proteins,^85^ aqueous complexes,^86–88^ and NaYF_4_:Yb/Er UCNPs,^1,2,5,89^ exhibit pronounced temperature-dependent changes in their optical or dynamical properties. The corresponding crossover temperature *T*_c_ typically falls within the 315–330 K range (Figure 3), near the minimum of water’s isothermal compressibility (Figure 2c). In panels a–c, changes in (i) the emission intensity ratio of Ag_2_S,^82^ (ii) the wavelength peak shift of CdTe QDs,^79^ and (iii) the collision-assisted interparticle energy transfer efficiency (ϕ_CA‑IPET_) between Yb^3+^ and Nd^3+^ ions in GdVO_4_:Yb/Nd nanoparticles^81^ were interpreted as signatures of a hydration-structure crossover, although density fluctuations were not explicitly discussed. In NaYF_4_:Yb/Er UCNPs (panels d–f), the steeper Brownian velocity increase above *T*_c_ is consistent with the reduction of LD motifs and the dominance of HD motifs.^2,89^ Because these observables (spectral shifts, emission intensity ratios, energy-transfer efficiencies, and Brownian velocity) are sensitive to hydration-layer structure, temperature-induced rearrangements in the LD/HD motif population manifest as discontinuities or bilinear trends. From the nanoprobe perspective, these temperature responses converge on a common origin: a crossover in the balance between LD and HD structural motifs within the hydration layer at *T*_c_.

**Figure 3:**
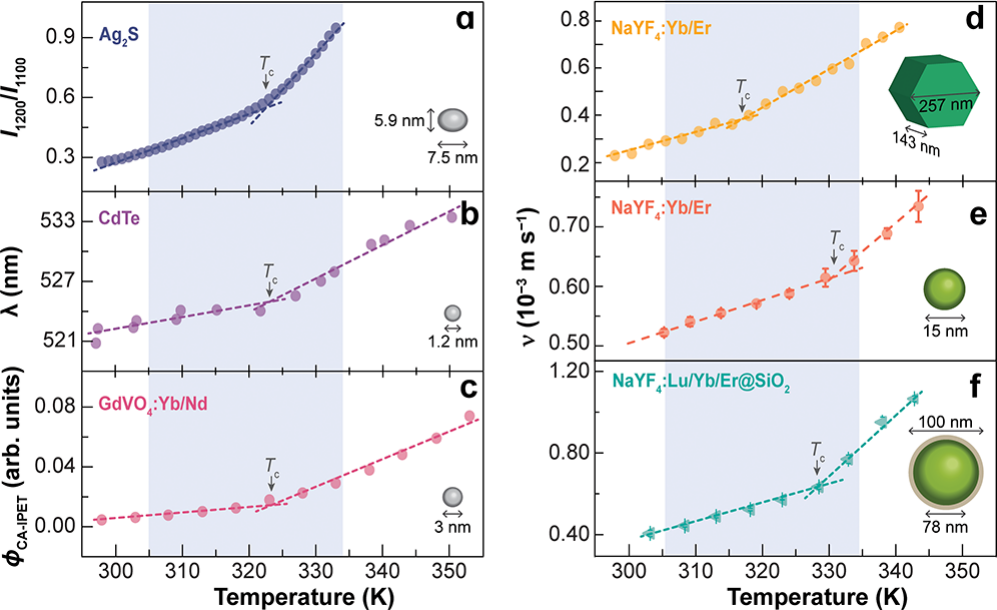
Several experimental observables exhibit bilinear temperature dependencies near the temperature range of the minimum of isothermal compressibility of water (Figure 2c), including (a) the emission intensity ratio (*I*_1200_/*I*_1100_) of silver sulfide (Ag_2_S) nanoparticles,^82^ (b) the shift of the maximum emission wavelength (λ) of cadmium telluride (CdTe) QDs dots,^79^ and (c) the collision-assisted interparticle energy transfer efficiency (ϕ_CA‑IPET_) between Yb^3+^ and Nd^3+^ ions in GdVO_4_:Yb/Nd nanoparticles.^81^ Brownian velocity (*v*) of NaYF_4_:Yb/Er UCNPs measured by (d) optical trapping experiments^89^ and (e, f) luminescence thermometry.^2^ The lines are linear fits to the data (*r* ^2^ > 0.984). The crossover temperatures (*T*_c_), determined from the intersection of the two linear regimes, are listed in Table 1. Insets show the morphology and size of each nanomaterial (not to scale).

Across these systems, *T*_c_ spans ≈315–330 K. This variability raises a central question: what determines the crossover temperature in the hydration layer of dispersed probes? Addressing this question requires separating the intrinsic structural response of hydration water from probe-dependent interfacial effects.

To this end, we examined how luminescent and dynamical observables evolve across nanoparticles with differing sizes, compositions, and surface terminations (Table 1). Each probe exhibits a distinct *T*_c_, indicating that the structural crossover is not governed by bulk thermodynamics alone but is instead shaped by interfacial interactions that modulate the balance of LD and HD motifs in the hydration layer.

Crucially, control measurements of nanofluid thermal diffusivity confirmed that *T*_c_ does not originate from changes in macroscopic heat-transport properties.^4^ Instead, luminescent nanoprobes respond selectively to structural rearrangements occurring in the few layers of water adjacent to their surface. Taken together, these observations establish *T*_c_ as an emergent, probe-dependent thermodynamic signature of the balance of LD/HD motifs in hydration water. The key challenge then becomes identifying the physical parameter that unifies these responses across disparate systems. As shown in the next section, the effective surface charge density of the probe provides such a framework, collapsing the observed variability in *T*_c_ into a single, physically intuitive description.

## 4 Effective Surface Charge as a Unifying Descriptor of the Crossover Temperature

Size, pH, and surface functionalization have each been proposed to influence *T*_c_ in hydration water, yet reported dependencies are often contradictory. Opposite pH trends have been reported^2,83,89,90^ (Figure 4a), while size-dependent shifts remain empirical and system-specific. This suggests that size and pH act indirectly, reflecting a more fundamental interfacial descriptor controlling the LD/HD balance in hydration water.^5^

**Figure 4:**
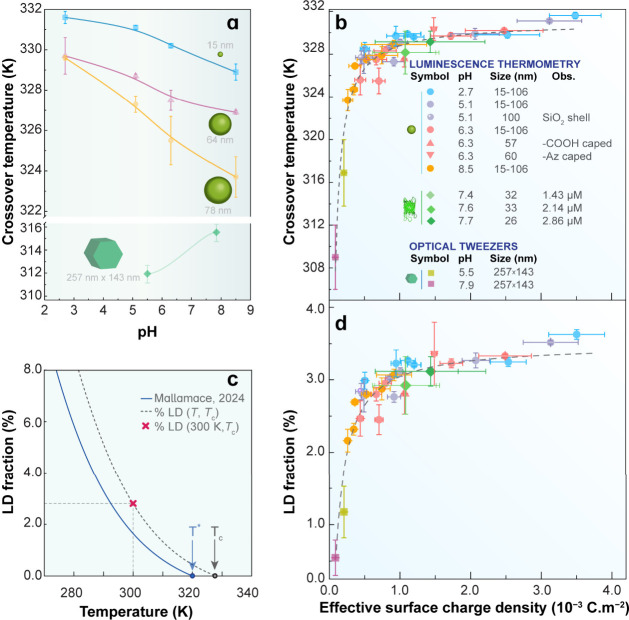
(a) Crossover temperatures *T*_c_ of aqueous suspensions of NaYF_4_:Yb/Er UCNPs as a function of pH, measured using optical trapping for the 257 nm × 143 nm sample^89^ and Brownian-velocity measurements^2^ for the remaining samples (15, 24, 52, 57, 60, 64, 78, 100, and 106 nm in diameter). Adapted from ref 2. Copyright 2024 American Chemical Society. (b) Experimental *T*_c_ values from refs 2 and 89 plotted against the effective surface charge density, *σ*_f_. Relative to ref 5, this panel additionally includes data from aqueous suspensions of NaYF_4_:Lu/Yb/Er@SiO_2_ ^2^ and EGFP.^6^ Adapted from ref 5. Copyright 2025 American Chemical Society. The dashed line is a fit to the combined data set (details in ref 5). (c) Schematic illustrating the procedure used to estimate the LD fraction at ambient pressure: the LD-population curve from Mallamace^54^ is adjusted (dashed line) to match the measured *T*_c_ (method in ref 5); the cross marks the corresponding LD fraction at 300 K. (d) Estimated LD fractions for the hydration layer of NaYF_4_:Yb/Er UCNPs and EGFP at 300 K as a function of *σ*_f_. Adapted from ref 5. Copyright 2025 American Chemical Society. The dashed line indicates the model prediction described in the reference.

A coherent explanation emerges when the effective surface charge density, *σ*_*f*_, is considered. The zeta potential, ζ, is the electrostatic potential at the slipping plane surrounding a colloidal particle, which encompasses both the nanoparticle surface and associated counterions. Within the Gouy–Chapman framework, ζ can be used to estimate an effective surface charge density that captures the net interfacial electrostatic strength:^91^

1 $$\sigma_{f}=\frac{\varepsilon_{0}\varepsilon_{r}\left|\zeta \right|}{r}$$

where *r* is the hydrodynamic radius (nanoprobe plus the slipping layer), and ε_0_ and ε_*r*_, are the vacuum and relative permittivities, respectively. Here, *σ*_f_ denotes the magnitude of the effective surface charge density (strictly non-negative because |ζ|, ε_0_, ε_*r*_, and *r* are positive). Although *σ*_f_ does not equal the true surface charge, molecular dynamics simulations show that in the low-charge regime relevant here (<16 × 10^–3^ C m^–2^), the two quantities scale linearly.^91^

When *T*_c_ is plotted against *σ*_f_, the previously scattered data for NaYF_4_:Yb/Er UCNPs^2,89^ collapse onto a single master curve spanning different particle sizes, pH values, surface chemistries, and measurement techniques (Figure 4b). This convergence demonstrates that the temperature at which LD motifs disappear is governed primarily by interfacial electrostatics rather than by nanoparticle size, surface termination, or pH alone. To further validate this connection, we compared two UCNP suspensions identical in size, concentration, and pH but functionalized with either COOH or azide groups. As anticipated, the sample with the larger *σ*_f_ exhibited a higher *T*_c_,^5^ confirming that interfacial electrostatics control the LD-HD structural crossover. Additional *T*_c_ values obtained from aqueous suspensions of SiO_2_-coated NaYF_4_:Lu/Yb/Er UCNPs^2^ and EGFP^6^ also fall on the same master curve. Notably, ligand-free and silica-coated UCNPs of similar size show comparable *T*_c_ values, consistent with their comparable surface charge densities.^2^ A similar consistency is observed across the three EGFP concentrations, which exhibit comparable *σ*_f_.^6^ Together, these results support *σ*_f_ as a dominant interfacial descriptor governing the LD/HD fluctuations in the hydration layer.

To connect *T*_c_ to hydration water structure, we estimated the LD fraction in the hydration layer of the NaYF_4_:Yb/Er UCNPs and EGFP at 300 K by combining the measured *T*_c_ values with a modified logistic model describing the temperature dependence of LD population, originally proposed by Hestand and Skinner^55^ and adapted by Mallamace (Figure 4c).^54^ The resulting LD fractions (2–5%) are lower than the ≈19% reported by Camisasca et al.^67^ for lysozyme hydration layer. Notably, the LD fraction increases systematically with *σ*_f_, indicating that stronger interfacial electric fields promote tetrahedral hydrogen bonding and stabilize LD motifs, thereby shifting *T*_c_ to higher temperatures.

Although these measurements probe hydration-layer dynamics, they can be compared with bulk-water simulations at ambient conditions, showing that externally applied electric fields stabilize LD motifs.^92^ The consistency between bulk simulations and our interfacial observations supports the interpretation that hydration water reflects bulk-derived LD/HD fluctuations whose local balance is modulated by electrostatic boundary conditions and confinement at the UCNP surface.

At high *σ*_f_, the LD fraction approaches saturation, reflecting the intrinsic upper bound set by the maximum LD population achievable in bulk water. Accordingly, the asymptotic behavior observed in Figure 4d indicates that increasing *σ*_f_ cannot generate additional LD motifs beyond the bulk-imposed limit, but instead progressively favors HD enrichment within the hydration shell. This saturation behavior is consistent with experimental^63^ and computational^67,93^ studies showing that protein hydration water is enriched in HD-like domains (or, equivalently, depleted in LD motifs) relative to the bulk, in agreement with independent observations that hydration water exhibits a higher density than bulk water.^61,64,94^ This trend implies that proteins with more strongly charged surfaces form more structured, LD-enriched hydration layers (Figure 4d) and may therefore exhibit enhanced thermal resistance (see next section). This hypothesis aligns with molecular dynamics simulations by Sterpone et al.,^95^ who demonstrated that the thermal resilience of hydration water is closely linked to the surface charge distribution of the biomolecule.

Altogether, *σ*_f_ emerges as a physically intuitive and experimentally accessible descriptor that reconciles previous inconsistencies in *T*_c_ across UCNPs and fluorescent proteins. By linking interfacial electrostatics to fluctuations between LD and HD motifs, this framework motivates testing whether nuclear quantum effects, which reshape hydrogen bonding and shift thermodynamic anomalies, produce corresponding shifts in the hydration-layer crossover temperature.

## 5 Nuclear Quantum Effects in Hydration Water and Protein Unfolding

Isotopic substitution provides an independent strategy to perturb hydration water structure and directly test how nuclear quantum effects influence the balance between LD and HD motifs.^36,96^ Heavy water (D_2_O) is well-known to increase protein melting temperatures by 2–9 K,^97,98^ indicating enhanced rigidity and thermal stability of the native fold.^99,100^ At the solvent level, deuteration modifies zero-point energies and strengthens hydrogen bonding, shifting thermodynamic anomalies to higher temperatures, including the minimum of the isothermal compressibility.^101–103^ These shifts are commonly discussed in terms of the displacement of the Widom line,^36,96,104–106^ which alters the temperature range of the fluctuations between LD and HD motifs.

A growing body of experimental and computational work indicates that protein stability is intimately coupled to the structural organization of hydration water. Proper protein function requires a stable first hydration layer,^107,108^ and disruptions to this layer have been associated with misfolding and neurodegenerative diseases such as Alzheimer’s and Parkinson’s.^109,110^

Neutron scattering, infrared spectroscopy, and molecular dynamics simulations show that the hydration water near the protein surface plays a major role in stabilizing the hydrogen bond networks.^60,111,112^ As temperature increases, the progressive depletion of LD motifs in the hydration shell is accompanied by faster hydrogen-bond dynamics, increased local density, and the emergence of HD-like coordination, ultimately destabilizing the native fold. Consistent with this picture, Mallamace et al.^60^ showed that above ≈320 K, hydrogen bonds in hydrated lysozyme break, destabilizing LD motifs and promoting unfolding. Oleinikova et al.^111^ reported a crossover in the heat capacity of hydration water at biosurfaces near 330 K, while Koizumi et al.^112^ observed that the collapse of the hydration layer precedes lysozyme unfolding, with *T*_c_ shifting between ≈318 K and ≈339 K depending on pH. Together, these studies support a unifying view in which protein unfolding coincides with a structural crossover in hydration layer, rather than arising solely from intrinsic changes in the protein backbone.

Within this framework, EGFP provides a particularly sensitive probe of hydration water organization, as both its fluorescence and native fold depend on a stable first hydration layer. Temperature-dependent fluorescence recovery shows that EGFP is more thermally stable in D_2_O than in H_2_O (Figure 5a), with both the onset of irreversible unfolding and the melting temperature shifting to higher values,^6^ consistent with prior reports of enhanced protein stability upon deuteration.^97–100^

**Figure 5:**
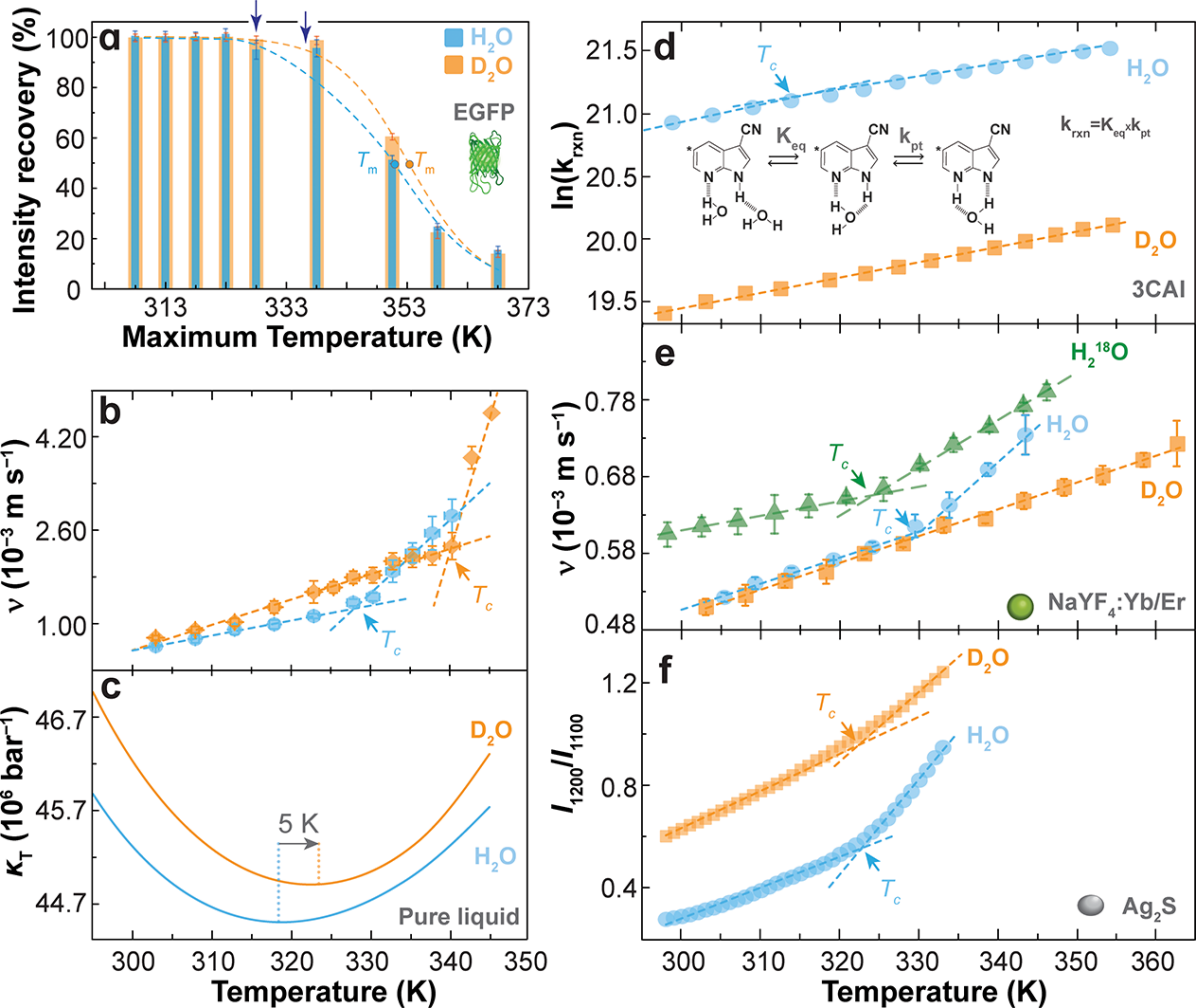
(a) Fluorescence intensity recovery of EGFP (2.86 μM) after heating–cooling cycles between 298 and 368 K in H_2_O and D_2_O. Solid lines are the best fit to the data using Boltzmann functions (*R* ^2^ > 0.988). Symbols indicate the melting temperature *T*_m_, and arrows mark the onset of irreversible unfolding. Adapted from ref 6. Copyright 2025 American Chemical Society. (b) Temperature-dependent Brownian velocity of EGFP in H_2_O and D_2_O. Dashed lines are linear fits to the two regimes (*r* ^2^ > 0.944), with *T*_c_ shifting to higher values in D_2_O. Adapted from ref 6. Copyright 2025 American Chemical Society. (c) Isothermal compressibility of pure H_2_O and D_2_O, showing an upward shift of the compressibility minimum in D_2_O. Data extracted and replotted from ref 101. (d) Temperature dependence of the excited-state proton-transfer rate constant (*k*_rxn_) of 3CAI.^87^ (e) Temperature-dependent Brownian velocity of the 15 nm NaYF_4_:Yb/Er UCNPs suspended in H_2_^18^O, H_2_O, and D_2_O. Adapted from ref 2. Copyright 2024 American Chemical Society. (f) Temperature-dependent emission intensity ratio *I*_1200_/*I*_1100_ of Ag_2_S nanoparticles.^82^ In panels (d-f), symbols represent experimental data and dashed lines are best linear fits (*r* ^2^ > 0.982). Nanoprobe morphologies and sizes are not shown to scale.

Brownian-velocity measurements directly reveal how nuclear quantum effects reshape hydration water fluctuations. In both H_2_O and D_2_O, EGFP exhibits a bilinear temperature dependence consistent with an LD-to-HD structural crossover. Crucially, *T*_c_ shifts upward from 328 ± 2 K in H_2_O to 338 ± 2 K in D_2_O (Figure 5b). This shift indicates that LD motifs persist to higher temperatures in heavy water, stabilizing the hydration layer and delaying the onset of EGFP unfolding.^36,100,102^ Circular-dichroism corroborates this behavior, with the melting temperature increasing from 352 ± 1 K in H_2_O to 356 ± 1 K in D_2_O.^6^ The thermodynamic basis for this shift is evident in the isothermal compressibility of H_2_O and D_2_O (Figure 5c). Because the compressibility minimum marks the temperature of maximal structural fluctuations, its upward displacement in D_2_O^113^ provides a plausible thermodynamic basis for the increase in *T*_c_ observed in the EGFP hydration layer. In this sense, EGFP translates a solvent-level quantum signature into a measurable nanoscale structural response.

In contrast, UCNPs and 3-cyano-7-azaindole (3CAI) do not detect an analogous isotopic shift (Figure 5d,e). For the 15 nm NaYF_4_:Yb/Er UCNPs, the Brownian velocity in H_2_O shows a clear bilinear dependence with a well-defined *T*_c_, whereas in D_2_O the temperature dependence becomes strictly linear, with no detectable crossover (Figure 5e). A similar disappearance of bilinearity is observed for the excited-state proton-transfer rate constant of 3CAI,^87^ which exhibits a crossover near 330 K in H_2_O but not in D_2_O (Figure 5d). Across both systems, heavy water suppresses the LD/HD signature that is clearly detectable in H_2_O.

The behavior of Ag_2_S nanoparticles is notably different. Although the original study did not explicitly identify the presence of a crossover temperature in D_2_O,^82^ the temperature dependence of the emission intensity ratio *I*_1200_/*I*_1100_ can be described either by a single linear relation or by two linear regimes, as observed in H_2_O (Figure 5f). The latter interpretation is consistent with sensitivity to LD/HD structural reorganizations of the hydration layer in both isotopic solvents. Within this bilinear description, the isotopic shift of the crossover temperature extracted from these data is comparable to the shift in the minimum of the isothermal compressibility between H_2_O and D_2_O (≈5 K, Figure 5c). However, the limited temperature range and experimental uncertainty preclude a definitive discrimination between single- and two-regime behavior, and dedicated measurements would be required to unambiguously resolve the presence and magnitude of an isotopic shift.

Heavy-oxygen water (H_2_^18^O) provides a critical control. Unlike H/D substitution, which strongly perturbs proton quantum fluctuations, ^18^O substitution introduces a relatively small change in nuclear mass and associated quantum effects and is therefore expected to leave the structure of hydration water largely unchanged, with at most a minor decrease in *T*_c_.^103,114,115^ Consistent with this expectation, the temperature-dependent Brownian velocity of NaYF_4_:Yb/Er UCNPs (15 nm diameter, 0.085% volume fraction) in H_2_^18^O yields *T*_c_ = 324 ± 1 K, close to that measured in H_2_O (Figure 5e and Table 1). These measurements, reported here for the first time, confirm that the dominant nuclear quantum contribution originates from hydrogen rather than oxygen.

The contrasting behavior between probes highlights a central open question: why do EGFP and Ag_2_S clearly sense the isotopic shift in *T*_c_, whereas UCNPs and 3CAI either fail to resolve a crossover in D_2_O or retain bilinear behavior? Although the underlying mechanism remains unresolved, these results demonstrate that the detectability of LD/HD fluctuations is inherently probe-dependent. It is important to note that isotopic nuclear quantum effects in D_2_O displace the Widom line^36^ and consequently shift *T*_c_ relative to H_2_O. In addition, interfacial confinement^116,117^ may modify how LD–HD fluctuations manifest, potentially preventing a distinct crossover from being resolved within the studied temperature window. Further investigations are required to determine whether the absence of bilinearity in D_2_O arises from a shifted *T*_c_ or from probe-dependent detectability limitations. Addressing this dependence will be essential for interpreting nanoscale measurements of water structure and for designing luminescent probes capable of capturing subtle quantum-induced modifications in hydration environments. Notably, as larger UCNPs exhibit lower *T*_c_ values,^2^ extending the study beyond the 15 nm particles investigated here may help determine whether size-dependent confinement effects can restore a detectable *T*_c_ in D_2_O.

## 6 Conclusions and Outlook

Together, the results discussed in this Account reinforce the view that fluctuations between low-density (LD) and high-density (HD) motifs extend into ambient conditions and leave measurable signatures in hydration environments. Luminescent nanoprobes, such as upconversion nanoparticles (UCNPs), and fluorescent proteins, provide a practical route to access these signatures. The crossover temperature, *T*_c_, identified from abrupt changes in optical or Brownian observables, correlates with the temperature at which LD motifs in the hydration shell become strongly depleted (or shrink below the probe’s effective length scale), yielding a probe-sensitive thermodynamic fingerprint of interfacial water reorganization.

Despite this progress, key questions remain unresolved. In particular, the pressure dependence and the extent to which LD/HD fluctuations persist across the phase diagram remain poorly constrained, largely because microscopic hydration structures are difficult to access under high pressure. Addressing this gap is essential for discriminating between competing pictures of water’s anomalous behavior, including the pressure-dependent “funnel of life” scenario^19^ and a nearly pressure-invariant singular temperature T*≈320 K, the so-called “magic point” at which water begins to exhibit its complex behavior (on cooling).^54,58,118,119^ Extending luminescence thermometry into controlled high-pressure environments, ideally while independently tracking structural metrics, will be especially valuable for establishing whether hydration-layer *T*_c_ follows bulk thermodynamic landmarks (e.g., Widom line or response-function extrema) or instead reflects more local, probe-mediated physics.

A deeper understanding of hydration water fluctuations is not only of fundamental interest but also of growing practical relevance. For example, Marques et al.^120,121^ reported that cisplatin alters the dynamic state of intracellular water in metastatic breast cancer cells, shifting it toward a more LDL-like hydrogen-bond network, suggesting that cellular water may act as a secondary target of chemotherapy. More broadly, these observations emphasize that interfacial water is not merely a passive background: its structural fluctuations can be tuned by charge, confinement, and surface chemistry, affecting colloidal stability, protein function, and potentially cellular physiology.

Looking forward, four directions appear particularly promising. First, expanding measurements into higher-pressure regimes (and across wider temperature windows) will help map hydration-water temperature crossover against the broader phase diagram. Second, developing ultrasensitive, well-calibrated interfacial probes (especially probes with independently tunable surface charge density and well-defined hydrodynamic size) will enable more quantitative structure–property relationships. Third, integrating luminescent nanothermometry with complementary structural and dynamical methods (neutron and X-ray scattering, ultrafast vibrational spectroscopies, and advanced simulations) will be critical for linking the structural crossover temperature *T*_c_ to specific microscopic markers of tetrahedrality, density fluctuations, and hydrogen-bond kinetics. Fourth, systematic benchmarking across probe classes (including UCNPs, QDs, semiconducting nanoparticles, and fluorescent proteins) should be undertaken to determine when isotopic substitution (H_2_O/D_2_O, with H_2_^18^O as a control) produces a resolvable shift in the hydration-layer crossover temperature. In addition to crossover detectability, future work should examine how isotopic substitution modifies the temperature dependence of ballistic dynamics, linking slope variations to thermophysical properties of the nanofluids. Establishing these “selection rules” for isotopic sensitivity (both in *T*_c_ shifts and in dynamical response) will be critical for interpreting nanoscale signatures of nuclear quantum effects and for engineering probes optimized to detect them.

Together, these advances should clarify the origin and universality of water’s interfacial structural density fluctuations and enable the rational design of materials and possibly therapeutic strategies that exploit water’s unique and still-unfolding complexity.

## References

[1] Brites C. D. S., Zhuang B. L., Debasu M. L., Ding D., Qin X., Maturi F. E., Lim W. W. Y., Soh D., Rocha J., Yi Z. G. (2020). Decoding a percolation phase transition of water at similar to 330
K with a nanoparticle ruler. J. Phys. Chem.
Lett..

[2] Maturi F. E., Raposo Filho R. S., Brites C. D. S., Fan J., He R., Zhuang B., Liu X., Carlos L. D. (2024). Deciphering density
fluctuations in the hydration water of Brownian nanoparticles via
upconversion thermometry. J. Phys. Chem. Lett..

[3] Guo Y. W., Maturi F. E., Brites C. D. S., Carlos L. D. (2024). Exploring green
fluorescent protein Brownian motion: Temperature and concentration
dependencies through luminescence thermometry. Adv. Phys. Res..

[4] Filho R. S. R., Brites C. D. S., He R., Liu X., Carlos L. D. (2025). Thermal
diffusivity of nanofluids: A simplified temperature oscillation approach. Phys. Fluids.

[5] Filho R. S. R., Brites C. D. S., Carlos L. D. (2025). Tuning water density fluctuations
with surface-charged colloidal nanoparticles probed by luminescence. J. Phys. Chem. Lett..

[6] Guo Y., Maturi F. E., Filho R. S. R., Brites C. D. S., Carlos L. D. (2025). Exploring
water beyond the solvent: Insights into density fluctuations and EGFP
unfolding via luminescence thermometry. J. Phys.
Chem. B.

[7] Chaplin M. (2006). Opinion -
Do we underestimate the importance of water in cell biology?. Nat. Rev. Mol. Cell Bio..

[8] Ball P. (2008). Water - An
enduring mystery. Nature.

[9] Pohorille A., Pratt L. R. (2012). Is water the universal
solvent for life?. Orig. Life Evol. Biosph..

[10] Bellissent-Funel M. C., Hassanali A., Havenith M., Henchman R., Pohl P., Sterpone F., van der Spoel D., Xu Y., Garcia A. E. (2016). Water determines
the structure and dynamics of proteins. Chem.
Rev..

[11] Brini E., Fennell C. J., Fernandez-Serra M., Hribar-Lee B., Luksic M., Dill K. A. (2017). How water’s
properties are
encoded in its molecular structure and energies. Chem. Rev..

[12] Ball P. (2017). Water is an
active matrix of life for cell and molecular biology. Proc. Natl. Acad. Sci. U.S.A..

[13] Errington J. R., Debenedetti P. G. (2001). Relationship
between structural order and the anomalies
of liquid water. Nature.

[14] Nilsson A., Pettersson L. G. M. (2015). The
structural origin of anomalous properties of liquid
water. Nat. Commun..

[15] Gallo P., Amann-Winkel K., Angell C. A., Anisimov M. A., Caupin F., Chakravarty C., Lascaris E., Loerting T., Panagiotopoulos A. Z., Russo J., Sellberg J. A., Stanley H. E., Tanaka H., Vega C., Xu L., Pettersson L. G. M. (2016). Water: A tale of two liquids. Chem. Rev..

[16] Chaplin, M. Water Structure and Science, 2017. Archived in the Internet Archive WaybackMachine. https://web.archive.org/web/*/http://www1.lsbu.ac.uk/water/water_anomalies.html/* (accessed 20–02–2026).

[17] Russo J., Akahane K., Tanaka H. (2018). Water-like anomalies as a function
of tetrahedrality. Proc. Natl. Acad. Sci. U.S.A..

[18] Stillinger F. H. (1980). Water revisited. Science.

[19] Pettersson, L. G. M. A two-state picture of water and the funnel of life. In Modern Problems of the Physics of Liquid Systems; Bulavin, L. A. , Xu, L. , Eds.; Springer Proceedings in Physics Vol. 223; Springer, 2019; pp 3–39. 10.1007/978-3-030-21755-6_1.

[20] Shadrack
Jabes B, Nayar D., Dhabal D., Molinero V., Chakravarty C. (2012). Water and
other tetrahedral liquids: Order, anomalies and solvation. J. Phys.: Condens. Matter.

[21] Sastry S., Austen Angell C. (2003). Liquid-liquid
phase transition in supercooled silicon. Nat.
Mater..

[22] Katayama Y., Mizutani T., Utsumi W., Shimomura O., Yamakata M., Funakoshi K. (2000). A first-order liquid-liquid phase
transition in phosphorus. Nature.

[23] Henry L., Mezouar M., Garbarino G., Sifré D., Weck G., Datchi F. (2020). Liquid-liquid transition
and critical
point in sulfur. Nature.

[24] Singh R. S., Biddle J. W., Debenedetti P. G., Anisimov M. A. (2016). Two-state thermodynamics
and the possibility of a liquid-liquid phase transition in supercooled
tip4p/2005 water. J. Chem. Phys..

[25] Tanaka H. (2000). Simple physical
model of liquid water. J. Chem. Phys..

[26] Shi R., Tanaka H. (2020). The anomalies and criticality
of liquid water. Proc. Natl. Acad. Sci. U.S.A..

[27] Yu Z. H., Shi R., Tanaka H. (2023). A unified description of the liquid structure, static
and dynamic anomalies, and criticality of TIP4P/2005 water by a hierarchical
two-state model. J. Phys. Chem. B.

[28] Poole P. H., Sciortino F., Essmann U., Stanley H. E. (1992). Phase-behavior of
metastable water. Nature.

[29] Palmer J. C., Martelli F., Liu Y., Car R., Panagiotopoulos A. Z., Debenedetti P. G. (2014). Metastable
liquid-liquid transition in a molecular
model of water. Nature.

[30] Palmer J. C., Poole P. H., Sciortino F., Debenedetti P. G. (2018). Advances
in computational studies of the liquid-liquid transition in water
and water-like models. Chem. Rev..

[31] Debenedetti P. G., Sciortino F., Zerze G. H. (2020). Second critical point in two realistic
models of water. Science.

[32] Weis J., Sciortino F., Panagiotopoulos A. Z., Debenedetti P. G. (2022). Liquid-liquid
criticality in the wail water model. J. Chem.
Phys..

[33] Sciortino F., Zhai Y., Bore S. L., Paesani F. (2025). Constraints on the
location of the liquid-liquid critical point in water. Nat. Phys..

[34] Angell C. A. (2008). Insights
into phases of liquid water from study of its unusual glass-forming
properties. Science.

[35] Angell C. A. (2014). Supercooled
water two phases?. Nat. Mater..

[36] Kim K. H., Spah A., Pathak H., Perakis F., Mariedahl D., Amann-Winkel K., Sellberg J. A., Lee J. H., Kim S., Park J. (2017). Maxima in the thermodynamic response and correlation
functions of deeply supercooled water. Science.

[37] Gallo P., Stanley H. E. (2017). Experiments provide evidence for two liquid phases
in supercooled water droplets. Science.

[38] Handle P. H., Loerting T., Sciortino F. (2017). Supercooled and glassy water: Metastable
liquid(s), amorphous solid(s), and a no-man’s land. Proc. Natl. Acad. Sci. U.S.A..

[39] Woutersen S., Ensing B., Hilbers M., Zhao Z., Angell C. A. (2018). A liquid-liquid
transition in supercooled aqueous solution related to the HDA-LDA
transition. Science.

[40] Kringle L., Thornley W. A., Kay B. D., Kimmel G. A. (2020). Reversible structural
transformations in supercooled liquid water from 135 to 245 K. Science.

[41] Kim K. H., Amann-Winkel K., Giovambattista N., Spah A., Perakis F., Pathak H., Parada M. L., Yang C., Mariedahl D., Eklund T. (2020). Experimental observation of the liquid-liquid transition
in bulk supercooled water under pressure. Science.

[42] Gallo P., Bachler J., Bove L. E., Bohmer R., Camisasca G., Coronas L. E., Corti H. R., de Almeida Ribeiro I., de Koning M., Franzese G., Fuentes-Landete V., Gainaru C., Loerting T., de Oca J. M. M., Poole P. H., Rovere M., Sciortino F., Tonauer C. M., Appignanesi G. A. (2021). Advances
in the study of supercooled water. Eur. Phys.
J. E.

[43] Suzuki Y. (2022). Direct observation
of reversible liquid-liquid transition in a trehalose aqueous solution. Proc. Natl. Acad. Sci. U.S.A..

[44] Amann-Winkel K., Kim K. H., Giovambattista N., Ladd-Parada M., Spaeh A., Perakis F., Pathak H., Yang C. L., Eklund T., Lane T. J. (2023). Liquid-liquid
phase
separation in supercooled water from ultrafast heating of low-density
amorphous ice. Nat. Commun..

[45] Kruger C. R., Mowry N. J., Bongiovanni G., Drabbels M., Lorenz U. J. (2023). Electron
diffraction of deeply supercooled water in no man’s land. Nat. Commun..

[46] Soper A. K., Ricci M. A. (2000). Structures of high-density and low-density
water. Phys. Rev. Lett..

[47] Huang C., Wikfeldt K. T., Tokushima T., Nordlund D., Harada Y., Bergmann U., Niebuhr M., Weiss T. M., Horikawa Y., Leetmaa M. (2009). The inhomogeneous
structure of water at ambient conditions. Proc.
Natl. Acad. Sci. U.S.A..

[48] Mallamace F., Corsaro C., Stanley H. E. (2013). Possible
relation of water structural
relaxation to water anomalies. Proc. Natl. Acad.
Sci. U.S.A..

[49] Shi R., Russo J., Tanaka H. (2018). Common microscopic structural origin
for water’s thermodynamic and dynamic anomalies. J. Chem. Phys..

[50] Lin C., Smith J. S., Sinogeikin S. V., Shen G. (2018). Experimental evidence
of low-density liquid water upon rapid decompression. Proc. Natl. Acad. Sci. U.S.A..

[51] Shi R., Tanaka H. (2020). Direct evidence in
the scattering function for the
coexistence of two types of local structures in liquid water. J. Am. Chem. Soc..

[52] Oka K., Shibue T., Sugimura N., Watabe Y., Tanaka M., Winther-Jensen B., Nishide H. (2021). Two states of water converge to one
state below 215 K. J. Phys. Chem. Lett..

[53] Foffi R., Sciortino F. (2024). Identification of local structures in water from supercooled
to ambient conditions. J. Chem. Phys..

[54] Mallamace F., Mallamace D. (2024). The water
bimodal inherent structure and the liquid-liquid
transition as proposed by the experimental density data. J. Chem. Phys..

[55] Hestand N. J., Skinner J. L. (2018). Perspective: Crossing the widom line in no man’s
land: Experiments, simulations, and the location of the liquid-liquid
critical point in supercooled water. J. Chem.
Phys..

[56] Novak N., Liang X. D., Kontogeorgis G. M. (2024). Prediction
of water anomalous properties
by introducing the two-state theory in SAFT. J. Chem. Phys..

[57] Muthachikavil A. V., Sun G., Peng B. L., Tanaka H., Kontogeorgis G. M., Liang X. D. (2024). Unraveling thermodynamic anomalies of water: A molecular
simulation approach to probe the two-state theory with atomistic and
coarse-grained water models. J. Chem. Phys..

[58] Mallamace F., Branca C., Broccio M., Corsaro C., Mou C. Y., Chen S. H. (2007). The anomalous behavior
of the density of water in the
range 30 K < T < 373 K. Proc. Natl. Acad.
Sci. U.S.A..

[59] Skinner L. B., Benmore C. J., Neuefeind J. C., Parise J. B. (2014). The structure of
water around the compressibility minimum. J.
Chem. Phys..

[60] Mallamace F., Mallamace D., Chen S. H., Lanzafame P., Papanikolaou G. (2021). Water thermodynamics
and its effects on the protein
stability and activity. Biophysica-Basel.

[61] Laage D., Elsaesser T., Hynes J. T. (2017). Water dynamics in the hydration shells
of biomolecules. Chem. Rev..

[62] Paschek D., Ludwig R. (2011). Specific ion effects
on water structure and dynamics
beyond the first hydration shell. Angew. Chem.,
Int. Ed..

[63] Lang X. Q., Shi L. X., Zhao Z. L., Min W. (2024). Probing the structure
of water in individual living cells. Nat. Commun..

[64] Persson F., Söderhjelm P., Halle B. (2018). The geometry of protein hydration. J. Chem.
Phys..

[65] Disalvo E.
A., Lairion F., Martini F., Tymczyszyn E., Frías M., Almaleck H., Gordillo G. J. (2008). Structural and functional
properties of hydration and confined water in membrane interfaces. Biochim. Biophys. Acta - Biomembranes.

[66] Merzel F., Smith J. C. (2002). Is the first hydration
shell of lysozyme of higher
density than bulk water?. Proc. Natl. Acad.
Sci. U.S.A..

[67] Camisasca G., Tenuzzo L., Gallo P. (2023). Protein hydration water: Focus on
low density and high density local structures upon cooling. J. Mol. Liq..

[68] Clark G. N. I., Hura G. L., Teixeira J., Soper A. K., Head-Gordon T. (2010). Small-angle
scattering and the structure of ambient liquid water. Proc. Natl. Acad. Sci. U.S.A..

[69] Soper A. K. (2010). Recent
water myths. Pure Appl. Chem..

[70] Duboué-Dijon E., Laage D. (2015). Characterization of the local structure in liquid water by various
order parameters. J. Phys. Chem. B.

[71] Soper A. K. (2019). Is water
one liquid or two?. J. Chem. Phys..

[72] Zhovtobriukh I., Besley N. A., Fransson T., Nilsson A., Pettersson L. G. M. (2018). Relationship
between X-ray emission and absorption spectroscopy and the local H-bond
environment in water. J. Chem. Phys..

[73] Maréchal Y. (2011). The molecular
structure of liquid water delivered by absorption spectroscopy in
the whole IR region completed with thermodynamics data. J. Mol. Struct..

[74] Morawietz T., Marsalek O., Pattenaude S. R., Streacker L. M., Ben-Amotz D., Markland T. E. (2018). The interplay of
structure and dynamics
in the Raman spectrum of liquid water over the full frequency and
temperature range. J. Phys. Chem. Lett..

[75] Taschin A., Bartolini P., Eramo R., Righini R., Torre R. (2013). Evidence of
two distinct local structures of water from ambient to supercooled
conditions. Nat. Commun..

[76] Wernet Ph., Nordlund D., Bergmann U., Cavalleri M., Odelius M., Ogasawara H., Naslund L. A., Hirsch T. K., Ojamae L., Glatzel P., Pettersson L. G. M., Nilsson A. (2004). The structure of the first coordination
shell in liquid water. Science.

[77] Köhler M. H., Barbosa R. C., da Silva L. B., Barbosa M. C. (2017). Role of the hydrophobic
and hydrophilic sites in the dynamic crossover of the protein-hydration
water. Physica A.

[78] Xu L., Mallamace F., Yan Z., Starr F. W., Buldyrev S. V., Eugene Stanley H. (2009). Appearance
of a fractional Stokes-Einstein relation
in water and a structural interpretation of its onset. Nat. Phys..

[79] Maestro L.M., Marques M.I., Camarillo E., Jaque D., Sole J. G., Gonzalo J.A., Jaque F., Valle J. C. D., Mallamace F., Stanley H.E. (2016). On the existence of two states in liquid water: Impact
on biological and nanoscopic systems. Int. J.
Nanotechnol..

[80] del
Valle J. C., Camarillo E., Martinez Maestro L., Gonzalo J. A., Arago C., Marques M., Jaque D., Lifante G., Sole J. G., Santacruz-Gomez K., Carrillo-Torres R. C., Jaque F. (2015). Dielectric anomalous
response of water at 60 °C. Philos. Mag..

[81] Labrador-Páez L., Jovanović D. J., Marqués M. I., Smits K., Dolić S. D., Jaque F., Stanley H. E., Dramićanin M. D., García-Solé J., Haro-González P. (2017). Unveiling molecular changes in water by small luminescent nanoparticles. Small.

[82] Munoz-Ortiz T., Abiven L., Marin R., Hu J., Ortgies D. H., Benayas A., Gazeau F., Castaing V., Viana B., Chaneac C. (2022). Temperature dependence
of water absorption in the biological
windows and its impact on the performance of Ag_2_S luminescent
nanothermometers. Part. Part. Syst. Charact..

[83] Catalan J., del Valle J. C. (2018). Molecule
1-methyl-5-nitroindoline probes the structural
change of liquid water with temperature. ACS
Omega.

[84] Catalan J., Gonzalo J. A. (2017). Liquid water changes its structure
at 43 °C. Chem. Phys. Lett..

[85] Mallamace F., Corsaro C., Mallamace D., Cicero N., Vasi S., Dugo G., Stanley H. E. (2015). Dynamical
changes in hydration water
accompanying lysozyme thermal denaturation. Front. Phys..

[86] D’Amico F., Bencivenga F., Gessini A., Masciovecchio C. (2010). Temperature
dependence of hydrogen-bond dynamics in acetic acid-water solutions. J. Phys. Chem. B.

[87] Cheng Y.-H., Yang H.-C., Chou P.-T. (2020). Could chemical
reaction at the molecular
level show distinction between two liquid-water states? Study of the
excited-state water-catalyzed proton transfer reaction provides a
clue. J. Phys. Chem. Lett..

[88] Labrador-Páez L., Montes E., Pedroni M., Haro-González P., Bettinelli M., Jaque D., Garcia-Solé J., Jaque F. (2018). Effect of H_2_O and D_2_O thermal anomalies on
the luminescence of Eu^3+^ aqueous complexes. J. Phys. Chem. C.

[89] Lu D., Labrador-Paez L., Ortiz-Rivero E., Frades P., Antoniak M. A., Wawrzynczyk D., Nyk M., Brites C. D. S., Carlos L. D., Garcıa Sole J. A., Haro-Gonzalez P., Jaque D. (2020). Exploring single-nanoparticle
dynamics at high temperature by optical tweezers. Nano Lett..

[90] Labrador-Páez L., Mingoes C., Jaque F., Haro-González P., Bazin H., Zwier J. M., Jaque D., Hildebrandt N. (2020). pH dependence
of water anomaly temperature investigated by Eu­(III) cryptate luminescence. Anal. Bioanal. Chem..

[91] Ge Z. P., Wang Y. (2017). Estimation of nanodiamond
surface charge density from zeta potential
and molecular dynamics simulations. J. Phys.
Chem. B.

[92] Cassone G., Martelli F. (2024). Electrofreezing of liquid water at
ambient conditions. Nat. Commun..

[93] Menendez C. A., Accordino S. R., Loubet N. A., Appignanesi G. A. (2024). Study of
protein hydration water with the V_4s_ structural index:
Focus on binding site description. J. Phys.
Chem. B.

[94] Barbosa R. D. C., Barbosa M. C. (2015). Hydration shell of the TS-Kappa protein:
Higher density
than bulk water. Physica A.

[95] Sterpone F., Bertonati C., Briganti G., Melchionna S. (2010). Water around
thermophilic proteins: The role of charged and apolar atoms. J. Phys.: Condens. Matter.

[96] Caupin F., Ragueneau P., Issenmann B. (2024). Isotope effect on the anomalies of
water: A corresponding states analysis. J. Chem.
Phys..

[97] Izzo V., Fornili S., Cordone L. (1975). Thermal denaturation
of B. subtilis
DNA in H_2_O and D_2_O observed by electron-microscopy. Nucleic Acids Res..

[98] Sasisanker P., Oleinikova A., Weingärtner H., Ravindra R., Winter R. (2004). Solvation
properties and stability of ribonuclease a in normal and deuterated
water studied by dielectric relaxation and differential scanning/pressure
perturbation calorimetry. Phys. Chem. Chem.
Phys..

[99] Cioni P., Strambini G. B. (2002). Effect of heavy water on protein flexibility. Biophys. J..

[100] Mendonça L., Steinbacher A., Bouganne R., Hache F. (2014). Comparative
study of the folding/unfolding dynamics of poly­(glutamic acid) in
light and heavy water. J. Phys. Chem. B.

[101] Millero F.
J., Lepple F. K. (1971). Isothermal
compressibility of deuterium
oxide at various temperatures. J. Chem. Phys..

[102] Soper A.
K., Benmore C. J. (2008). Quantum
differences between heavy
and light water. Phys. Rev. Lett..

[103] Kutus B., Shalit A., Hamm P., Hunger J. (2021). Dielectric
response of light, heavy and heavy-oxygen water: Isotope effects on
the hydrogen-bonding network’s collective relaxation dynamics. Phys. Chem. Chem. Phys..

[104] Wang Z., Ito K., Leao J. B., Harriger L., Liu Y., Chen S. H. (2015). Liquid-liquid phase
transition and its phase diagram
in deeply-cooled heavy water confined in a nanoporous silica matrix. J. Phys. Chem. Lett..

[105] Zhang Y., Faraone A., Kamitakahara W. A., Liu K. H., Mou C. Y., Leao J. B., Chang S., Chen S. H. (2011). Density hysteresis of heavy water confined in a nanoporous
silica matrix. Proc. Natl. Acad. Sci. U.S.A..

[106] Kimmel G. A. (2024). Isotope
effects in supercooled H_2_O and D_2_O and a corresponding-states-like
rescaling of the temperature
and pressure. J. Chem. Phys..

[107] Rupley J. A., Yang P. H., Tollin G. (1980). Water-protein
interactions. Biophys. J..

[108] Rupley J. A., Careri G. (1991). Protein hydration and
function. Adv. Protein Chem..

[109] Chiti F., Dobson C. M. (2009). Amyloid formation
by globular proteins
under native conditions. Nat. Chem. Biol..

[110] Selkoe D. J. (2003). Folding
proteins in fatal ways. Nature.

[111] Oleinikova A., Brovchenko I., Singh G. (2010). The temperature dependence
of the heat capacity of hydration water near biosurfaces from molecular
simulations. Europhys. Lett..

[112] Koizumi M., Hirai H., Onai T., Inoue K., Hirai M. (2007). Collapse of the hydration shell of
a protein prior to thermal unfolding. J. Appl.
Crystallogr..

[113] Eltareb A., Lopez G. E., Giovambattista N. (2025). Isotope-substitution
effects on the thermodynamic, dynamic, and structural properties of
water: H_2_O, HDO, D_2_O, and T_2_O. J. Phys. Chem. B.

[114] Berger A., Ciardi G., Sidler D., Hamm P., Shalit A. (2019). Impact of nuclear quantum effects
on the structural
inhomogeneity of liquid water. Proc. Natl. Acad.
Sci. U.S.A..

[115] Steinberg P. Y., Corti H. R. (2025). Volumetric properties of H_2_
^18^O and its mixtures with ordinary water. J. Phys. Chem. Ref. Data.

[116] Dupertuis N., Tarun O. B., Lutgebaucks C., Roke S. (2022). Three-dimensional confinement of water: H_2_O exhibits long-range
(> 50 nm) structure while D_2_O does not. Nano Lett..

[117] Kim Y., Ding H. R., Zheng Y. B. (2022). Investigating
water/oil interfaces
with opto-thermophoresis. Nat. Commun..

[118] Mallamace F., Mallamace D., Mensitieri G., Chen S. H., Lanzafame P., Papanikolaou G. (2021). The water
polymorphism and the liquid-liquid transition from transport data. Physchem-Basel.

[119] Ginzburg V. V., Fazio E., Corsaro C. (2023). Combined description
of the equation of state and diffusion coefficient of liquid water
using a two-state Sanchez-Lacombe approach. Molecules.

[120] Marques M. P. M., Batista de Carvalho A.
L. M., Mamede A. P., Rudic S., Dopplapudi A., Garcia Sakai V., Batista de Carvalho L. A. E. (2020). Intracellular water as a mediator
of anticancer drug action. Int. Rev. Phys. Chem..

[121] Marques M. P. M., de Carvalho A. L. M.
B., Martins C. B., Silva J. D., Sarter M., García Sakai V., Stewart J. R., de Carvalho L. A. E.
B. (2023). Cellular dynamics as
a marker of normal-to-cancer transition in human cells. Sci. Rep..

